# H19 potentiates let-7 family expression through reducing PTBP1 binding to their precursors in cholestasis

**DOI:** 10.1038/s41419-019-1423-6

**Published:** 2019-02-18

**Authors:** Li Zhang, Zhihong Yang, Wendong Huang, Jianguo Wu

**Affiliations:** 1 Department of Molecular and Cellular Oncology, University of Texas MD Anderson Cancer Center, Houston, TX 77030 USA; 2 Division of Gastroenterology and Hepatology, Department of Medicine, Indiana University School of Medicine, Indianapolis, IN 46202 USA; 3 Department of Diabetes Complications and Metabolism, Diabetes and Metabolism Research Institute, Beckman Research Institute, City of Hope National Medical Center, Duarte, CA 91010 USA; 4 Veterans Affairs Connecticut Healthcare System, West Haven, CT 06516 USA; 5 Department of Physiology and Neurobiology, Institute for Systems Genomics, University of Connecticut, Storrs, CT 06269 USA

## Abstract

Cholestasis induces the hepatic long non-coding RNA H19, which promotes the progression of cholestatic liver fibrosis. However, microRNAs that are dysregulated by H19 during cholestasis remain elusive. Using miRNA-sequencing analysis followed by qPCR validation, we identified marked upregulation of eight members of the let-7 family in cholestatic livers by bile duct ligation (BDL) and H19 overexpression. In particular, the expression of let-7a-1/7d/7f-1 was highly induced in H19-BDL livers but decreased in H19KO-BDL livers. Interestingly, H19 decreased the nuclear let-7 precursors as well as the primary transcripts of let-7a-1/7d/7f-1 levels in BDL mouse livers. Bioinformatics, RNA pull-down, and RNA immunoprecipitation (RIP) assays revealed that the crucial RNA-binding protein polypyrimidine tract-binding protein 1 (PTBP1), an H19 interaction partner, interacted with the precursors of let-7a-1 and let-7d and suppressed their maturation. Both PTBP1 and let-7 expression was differentially regulated by different bile acid species in hepatocyte and cholangiocyte cells. Further, H19 negatively regulated PTBP1’s mRNA and protein levels but did not affect its subcellular distribution in BDL mouse livers. Moreover, we found that H19 restrained but PTBP1 facilitated the bioavailability of let-7 miRNAs to their targets. Taken together, this study revealed for the first time that H19 promoted let-7 expression by decreasing PTBP1’s expression level and its binding to the let-7 precursors in cholestasis.

## Introduction

The imprinted oncofetal long non-coding RNA (lncRNA) H19 is one of the first identified imprinted lncRNAs and is predominantly distributed in the cytoplasm of cells^1,2^. Due to the methylation modifications within the differentially methylated region (DMR) of *H19* promoters, H19 is only transcribed from the maternally inherited allele while the paternal *H19* allele is not expressed^3^. The aberrant H19 expression has been frequently linked to human Beckwith-Wiedemann syndrome and Silver-Russell syndrome^4,5^. Intriguingly, H19 maintains a high expression level in embryogenesis but is barely detectable in most of the tissues after birth except muscle and heart, implying a crucial role in mammal development and growth^3^. Although extensive studies have revealed important roles of H19 in various cancers^6^, the regulation of H19 in human liver diseases is largely uncovered. Emerging evidence shows that reactivation of H19 expression exacerbates cholestatic liver fibrosis^7^ and the development of fatty liver^8^. Phenotypically, the high induction of H19 expression is observed in human cirrhotic livers^9^. Despite these recent advances, the downstream molecular networks of H19 in liver pathogenesis remain elusive.

The polypyrimidine tract-binding protein 1 (PTBP1, also known as PTB or heteronuclear ribonucleoprotein (hnRNP) I) is an RNA-binding protein and regulates precursor mRNA (pre-mRNA) splicing, alternative splicing events, and mRNA stability^10^. PTBP1 has been implicated in different liver diseases^8^. PTBP1 complexes with heterogeneous nuclear RNA in the nucleus to regulate pre-mRNA processing and other aspects of mRNA metabolism and transport. PTBP1 has been reported to associate with multiple lncRNAs. For instance, maternally expressed 3 (MEG3), another lncRNA, binds to PTBP1 to control small heterodimer partner mRNA stability and cholestatic liver injury^11^, whereas H19 binds PTBP1 and reprograms hepatic lipid homeostasis^8^. In most mammals, there are two tissue-specific isoforms of PTBP. PTBP1 is widely expressed, while PTBP2 (also called nPTB or brPTB) is mainly expressed in neurons and testis^12^. The PTBP proteins preferentially bind CU tracts (e.g., UCUUC and CUCUCU, located within a polypyrimidine-rich context in RNAs). Because all four repeats of quasi-RNA recognition motif domains in PTBP can bind RNAs, it is difficult to define one RNA consensus sequence and to identify RNA targets of PTBP^13^. The interaction between PTPB1 with miRNAs has been noticed^14^, but the exact role of how PTPB1 regulates miRNA expression remains to be determined.

Let-7 belongs to a family of miRNAs required for development timing, tumor suppression, and metabolism regulation^15^. To generate a let-7 miRNA, a primary transcript (pri-let-7) is transcribed by RNA polymerase II and then subsequently processed. Pri-let-7 is cleaved by the microprocessor complex, composed of Drosha and its cofactor DGCR8, to produce precursor let-7 (pre-let-7) in the nucleus. Pre-let-7 is then exported into the cytoplasm and cleaved into an ~22-nucleotide duplex by Dicer complex, followed by unloading into argonaute (AGO) proteins that are essential components of the RNA-induced silencing complex (RISC)^15,16^. In addition to these basic processing factors, the biogenesis of let-7 is also tightly regulated by other cellular factors, such as the RNA-binding proteins (RBPs) LIN28A/B and DIS3L2^17,18^. Dysregulation of let-7 processing contributes to multiple pathological processes including cholestatic liver diseases^19^.

The goal of this study is to identify aberrant miRNAs that are regulated by H19 in cholestatic liver fibrosis. In this study, we determined the role of H19 and its binding protein PTPB1 in the expression and bioavailability of a cluster of let-7 miRNAs in cholestasis. The results show that H19 represses PTPB1 expression in cholestatic mouse livers, which is permissive to let-7 maturation from precursors. We also reveal that the bioavailability of let-7 miRNAs is suppressed by H19 but facilitated by PTPB1.

## Materials and methods

### Mouse models

*H19*-deficient mice were described previously^7^. Because *H19* is a paternally imprinted gene, maternal *H19*-deleted mice were used for experiments and paternal *H19*-deleted mice were used as negative controls. Mice were fed a standard rodent chow diet (#2918, Teklad global 18% protein rodent diets; Envigo Bioproducts, Inc) with free access to water and maintained in a 12 h light/dark cycle (light on 6 a.m. to 6 p.m.), temperature-controlled (23 °C), and pathogen-free facility. In vivo experiments were performed on mice (*n* = 3–5) at the age of 6 weeks unless stated otherwise. Considering sex as a biological variable does not affect the induction of cholestasis after bile duct ligation (BDL), only male mice were involved in the study to reduce animal use. For in vivo viral transduction^20^, mice (C57BL/6J, Jackson Laboratory, Bar Harbor, ME) were injected via tail vein with purified adeno-associated viral vector serotype 8 (AAV8) containing a liver‐specific thyroxine‐binding globulin promoter driving *H19* gene expression (5 × 10^10^ virus particles/mouse). The operation of common BDL has been described previously^21^. All experiments were performed in accordance with the guidelines and regulations approved by the Institutional Animal Care and Use Committee at the University of Connecticut.

### MicroRNA-sequencing analysis

MicroRNA-sequencing (miR-seq) was conducted at the Division of Experimental and Translational Genetics, Children’s Mercy Hospital (Kansas City, MO). A total of 14 livers were subjected to miR-seq analysis (Null-sham, *n* = 3; Null-BDL, *n* = 4; H19-sham, *n* = 3; and H19-BDL, *n* = 4). The read counts of miR-seq were used as criteria to compare miRNA levels. Quantitative PCR (qPCR) validation of miRNA expression was conducted in a new cohort of male mice subjected to sham or BDL (*n* = 5/group).

### Cell culture

Cell culture procedures for human hepatocellular carcinoma (HCC) cell line Huh7 and mouse HCC cell line Hepa1 have been reported previously and maintained in Dulbecco’s modified Eagle’s medium with 100 μg/ml streptomycin, 100 U/ml penicillin, and 10% fetal bovine serum^22,23^. Cell culture procedures for mouse small cholangiocytes (MSCs) and mouse large cholangiocytes (MLCs) were described previously^7^. Mouse primary hepatocytes were isolated and cultured as described previously^24^.

### Chemicals, plasmids, antibodies, and enzyme-linked immunosorbent assay kits

Dimethyl sulfoxide (DMSO), cholic acid (CA), chenodeoxycholic acid (CDCA), lithocholic acid (LCA), ursodeoxycholic acid (UDCA), and taurocholic acid (TCA) were purchased from Sigma-Aldrich (St. Louis, MO) and the cellular treatment was described previously^7^. Plasmids including pcDNA3-pri-let-7a-1 (#51377), pcDNA3-pri-let-7d (#51379), and psiCHECK2-let-7 4× (#20930) were purchased from Addgene. The expression plasmids for pri-let-7a-1 and pri-let-7d^25^, and for PTBP1^11^, and the let-7 luciferase plasmid^26^ have been described previously. The following antibodies were used for RNA precipitation and/or western blotting: PTBP1 (cat. 32-4800; Thermo Fisher Scientific, Waltham, MA), β-actin (cat. A-1978; Sigma-Aldrich), Lamin A/C (sc-7292; Santa Cruz Biotechnology, Santa Cruz, CA), Cyclophilin A (ab58144; Abcam, Cambridge, UK), AGO2 (cat. 04-642; EMD Millipore, Burlington, MA), HMGA2 (#8179; Cell Signaling Technology, Danvers, MA), STAT3 (#9139s; Cell Signaling Technology), TLR4 (sc-293072; Santa Cruz Biotechnology), and a-Tubulin (sc-5286; Santa Cruz Biotechnology). Interleukin (IL)-6 enzyme-linked immunosorbent assay (ELISA) kit (Cat. 88-7064-22) was from Thermo Fisher Scientific. IL-10 ELISA kit (Cat. M1000B) was from R&D Systems (Minneapolis, MN). The ELISA assays were performed according to the manufacturers’ instructions.

### Reverse transcription-qPCR

RNAs were prepared using Trizol reagent (Thermo Fisher Scientific)^27^. For mature (miR) and precursor miRNAs (pre-miRNA), 1 μg of RNA was reversely transcribed using a miScript II RT Kit (QIAGEN, Germantown, MD). Mature and pre-miRNAs were analyzed by a miScript Green PCR Kit (QIAGEN) using miScript Primer Assays for mm-let-7a-5p, mm-let-7d-5p, mm-let-7f-5p, hsa-let-7a-5p, and hsa-let-7d-5p, and miScript Precursor Assays for precursors of mm-let-7a-1, mm-let-7d, and mm-let-7f-1 (QIAGEN). For let-7 target genes and pri-let-7, total RNAs were reversely transcribed using a High-Capacity cDNA Reverse Transcription Kit with RNase Inhibitor (#4374967; Thermo Fisher Scientific) and qPCR was performed using the SsoAdvanced™ Universal SYBR® Green Supermix (#1725275; Bio-Rad, Hercules, CA). The control RNA Snord68 (also known as MBII-202) was quantitated using TaqMan™ MicroRNA Assay (ID: 001232) and TaqMan™ Universal PCR Master Mix (Cat. 4324018) purchased from Thermo Fisher Scientific.

### Preparation of RNAs from nuclear and cytoplasmic extracts

As described previously, the nuclear and cytoplasmic fractions of mouse liver tissues were prepared using a hypotonic buffer and a high-salt buffer that were supplemented with RNase inhibitor (1 U/µl)^22^. The fractions were immediately extracted for RNAs using Trizol reagent.

### Biotinylated RNA pull-down assay and RNA immunoprecipitation assay

A biotinylated RNA pull-down assay was performed as described previously^11^. Briefly, whole-cell lysates were incubated fully with purified biotinylated RNA probes that were synthesized via in vitro transcription. RNA–protein complexes were further isolated by Streptavidin Sepharose High-Performance beads (GE Healthcare, Marlborough, MA). The recruited proteins were detected by western blotting with antibodies. For RNA immunoprecipitation (RIP), anti-PTBP1 antibody or mouse immunoglobulin G (Sigma) was incubated with ultraviolet-crosslinked cell lysate for 2 h with gentle shaking. Protein A/G agarose beads were added to recruit RNA–protein complexes. RNAs associated with PTBP1 were recovered with Trizol-chloroform and analyzed by reverse transcription-qPCR.

### Other standard methods

Western blotting, RNA extraction from cells and liver tissues, qPCR, transient transfection, and luciferase reporter assays were performed as described previously^22,28–31^. The primers were listed in Supplementary Table 2. Additional results were included in the Supplementary Figure files.

### Statistical analysis

All cell-based in vitro experiments were performed in triplicate and repeated at least two times. All animal-based in vivo experiments were performed with various animals (*n* = 3–5) per group based on an estimated statistical power for over 80% possibility to find significant difference. The data were displayed as the mean values ± standard error of the mean^32^. Statistical analysis was carried out using the Student’s *t* test. *P* < 0.05 was considered statistically significant.

## Results

### Hepatic let-7 family is markedly induced by H19 in BDL-induced cholestasis

**Fig. 1:**
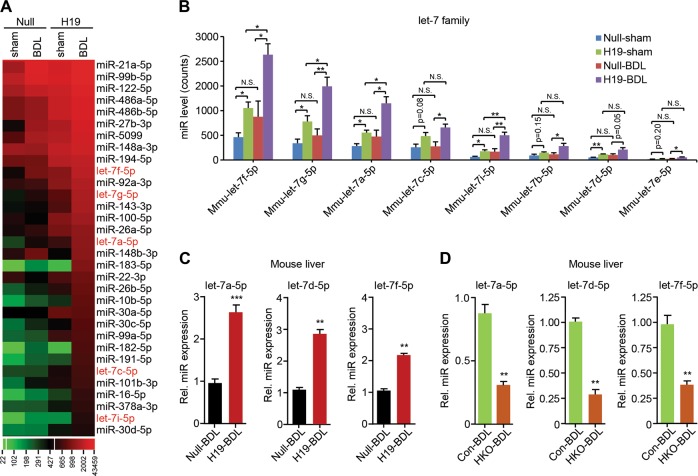
H19 increases mature let-7a-5p/7d-5p/7f-5p levels in mouse bile duct ligation (BDL) model. **a** The heat map showing differential hepatic expression of let-7 family members. Wild-type (WT) mice were injected with either an empty adeno-associated viral vector serotype 8 (AAV8) virus (Null) or an *H19*-expression AAV8 virus (H19) for 1 month to achieve stable gene expression. Then, these mice were subjected to either sham or BDL surgery for 1 week. Livers were then collected and RNA was extracted and subjected to microRNA-sequencing (miR-seq). The average count numbers of top 32 miRNAs in each group were plotted. **b** The count numbers of individual let-7 family members from the miR-seq results were graphed. **c** Quantitative PCR (qPCR) of let-7a-5p, let-7d-5p, and let-7f-5p in Null-BDL and H19-BDL mouse livers (*n* = 5 mice/group). **d** qPCR of let-7a-5p, let-7d-5p, and let-7f-5p in BDL-operated paternal *H19* knockout (con) and maternal *H19* knockout (HKO) mouse livers (*n* = 5 mice/group). Data were presented as the mean ± SEM from triplicate assays. *P < 0.05, **P < 0.01, ***P < 0.001; N.S. no significance

As described previously, a scramble control AAV8 virus (Null) or an H19-expressing AAV8 virus (H19) were injected into wild-type (WT) mice via the tail vein to generate an H19 overexpression mouse model^7^. We then performed BDL for 7 days to induce cholestasis in these mice. As expected, hepatic *H19* RNA level was elevated in H19-BDL vs Null-BDL mice (Fig. S1A). To profile H19-regulated miRNAs in cholestasis, we performed miR-seq in Null-BDL and H19-BDL livers, as well as in sham-operated livers (Null-sham and H19-sham). The hepatic expression of 32 miRNAs was robustly induced by H19 overexpression either in sham- or BDL-operated mice (Fig. 1a). Of note, several let-7 family members with higher basal expression including mmu-let-7f-5p, mmu-let-7g-5p, mmu-let-7a-5p, and mmu-let-7c-5p, showed up among these upregulated miRNAs (H19-sham vs Null-sham; H19-BDL vs Null-BDL) (Fig. 1a, b and Supplementary EXCEL file). In addition, four let-7 family members of lower abundance (mmu-let-7i-5p, mmu-let-7b-5p, mmu-let-7d-5p, and mmu-let-7e-5p) were also significantly induced in H19-BDL livers, comparing with Null-BDL mice (Fig. 1b). These results demonstrate that exogenous H19 overexpression potentiates let-7 family expression. Although the hepatic endogenous H19 expression is elevated by BDL^7,33^, unexpectedly, the eight let-7 miRNAs were plausibly but not significantly induced by BDL in Null-mice (Null-BDL vs Null-sham); however, the expression of mmu-let-7f-5p, mmu-let-7g-5p, mmu-let-7a-5p, and mmu-let-7i-5p was significantly increased by BDL in H19 mice (H19-BDL vs H19-sham) (Fig. 1b), suggesting that different from the incompetence of BDL-induced H19 (7 days), long-term overexpressed H19 (5 weeks) in the liver^7^ might sensitize cellular context to predispose cells to BDL induction of let-7. We further performed qPCR with specific primers to validate the expression of the above let-7 family members in a new cohort of Null and H19 mice, which were subjected to BDL. Let-7a-5p, let-7d-5p, and let-7f-5p displayed significantly higher expression levels in H19-BDL livers vs Null-BDL livers (Fig. 1c). Unexpectedly, the expression of let-7b-5p, let-7c-5p, let-7i-5p, and let-7g-5p was not induced in H19-BDL vs Null-BDL mice (Fig. S1B), suggesting animal individual variations from two different cohorts of mice. Of particular note, the expression levels of let-7a-5p/7d-5p/7f-5p were markedly elevated in primary hepatocytes isolated from H19-BDL compared to Null-BDL livers, suggesting that H19 activated let-7 family expression in hepatocytes during cholestasis (Fig. S1C). We also compared the hepatic expression of let-7a-5p/7d-5p/7f-5p in H19-sham and Null-sham mice using qPCR. The results support that H19 potentiates let-7 expression (Fig. S1D).

We therefore asked whether loss of H19 suppressed let-7 family expression during cholestasis. As expected, the hepatic expression of let-7a-5p/7d-5p/7f-5p was suppressed in maternal *H19* knockout-BDL mice (HKO-BDL) vs paternal *H19* knockout-BDL mice (Con-BDL) (Fig. 1d and S1E). Levels of let-7a-5p/7d-5p/7f-5p in the primary hepatocytes from HKO-BDL mice were also much lower than those from the Con-BDL (data not shown).

Taken together, the above results demonstrated that H19 positively regulated hepatic let-7 family expression in cholestatic mice.

### Bile acid species exhibit differential potency to regulate let-7 expression

**Fig. 2:**
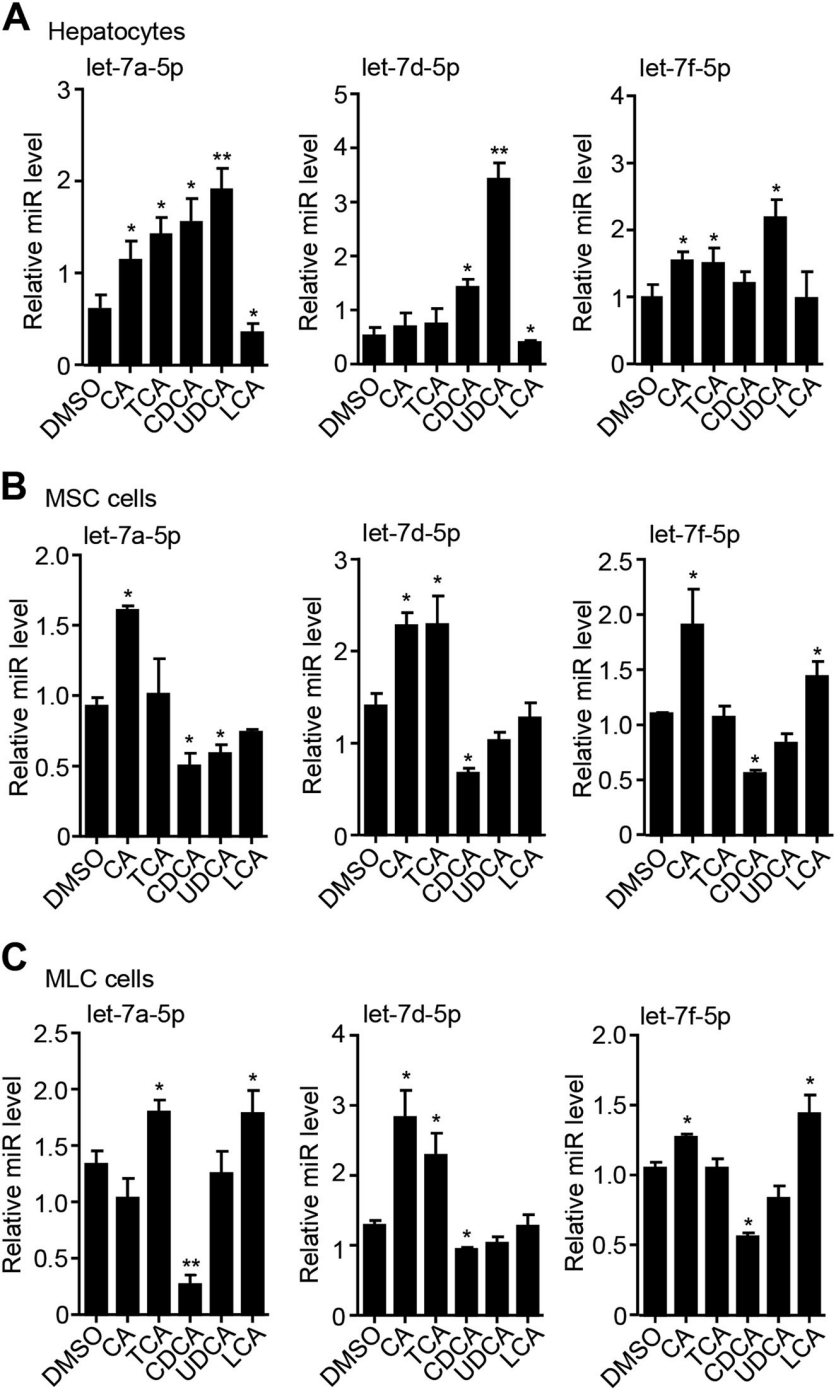
The expression of let-7a-5p/7d-5p/7f-5p is regulated by bile acids in hepatic cells. **a**–**c** Quantitative PCR of let-7a-5p, let-7d-5p, and let-7f-5p in mouse primary hepatocytes (**a**), small cholangiocytes (MSCs) (**b**), and large cholangiocytes (MLCs) (**c**). Cells were treated with cholic acid (CA, 100 µM), taurocholic acid (TCA, 100 µM), chenodeoxycholic acid (CDCA, 100 µM), ursodeoxycholic acid (UDCA, 100 µM), or lithocholic acid (LCA, 10 µM) for 24 h. Data were shown as mean ± SEM from triplicate assays. *P < 0.05, **P < 0.01 vs dimethyl sulfoxide

BDL-induced cholestasis causes the disruption of bile flow thus the accumulation of bile acids (BAs) in the liver, which deteriorates biliary epithelia (cholangiocytes) to promote the development of biliary hyperplasia, intrahepatic inflammatory response, and biliary liver fibrosis. In order to determine whether BAs regulate let-7 family expression, mouse primary hepatocytes were isolated from WT mice and treated with various BAs. Since let-7a-5p/7d-5p/7f-5p were significantly upregulated by H19 in cholestatic mouse livers, we focused on the expression of these three let-7 family members. Overall, the expression of let-7a-5p, let-7d-5p, and let-7f-5p was induced by different BAs to various extents (Fig. 2a). UDCA showed the highest potency to induce all three let-7 members, whereas LCA appeared to moderately repress let-7a-5p and let-7d-5p expression.

We also examined the effects of BAs on let-7 expression in MSCs and MLCs. In MSC cells, CA exhibited the highest potency to induce let-7a-5p, let-7d-5p, and let-7f-5p expression, whereas CDCA inhibited all three let-7 expression (Fig. 2b). In MLC cells, TCA and LCA induced let-7a-5p; CA and TCA induced let-7d-5p; CA and LCA induced let-7f-5p, whereas CDCA inhibited all three let-7 members (Fig. 2c). Because these BAs showed no effects on H19 promoter luciferase reporter activities in MSC and MLC cells (Fig. S2A), we postulated that the induction of let-7a-5p/7d-5p/7f-5p in H19-BDL mice might be presumably not due to a direct activation of H19 transcription by BAs.

### H19 promotes maturation of let-7 miRNAs in BDL-induced cholestasis

Let-7 family members are expressed either independently or as clusters from different genomic location. The biogenesis of let-7 miRNA is a complex process involving two key processing steps, i.e. from primary-miRNA (pri-miR) to the pre-miR and from pre-miR to the miR^15^. The murine let-7a-5p/7d-5p/7f-5p are derived from the let-7a-1/7d/7f-1 cluster in chromosome 13 (Supplementary Table 1); the pri-miRNAs from this cluster occupy a substantial portion of all let-7 primary transcripts (~24% in human)^34^.

**Fig. 3:**
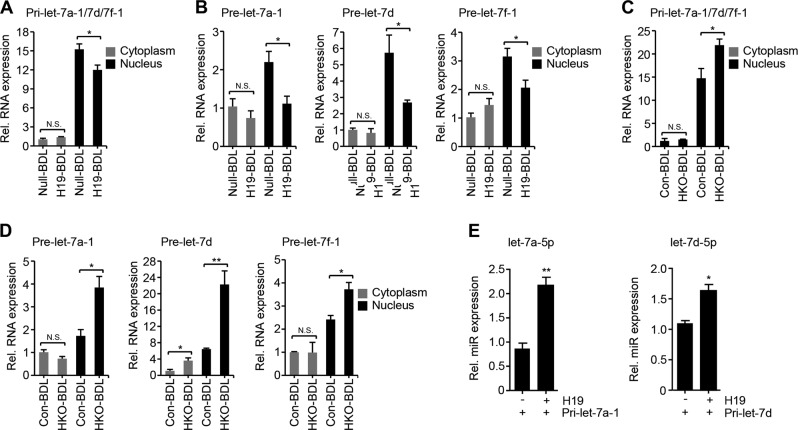
H19 regulates the levels of let-7 primary transcripts and precursors in cholestatic mouse livers. **a**, **b** Quantitative PCR (qPCR) of the expression of pri-let-7a-1/7d/7f-1 (**a**) and pre-let-7a-1/7d/7f-1 (**b**) in the cytoplasmic and nuclear extracts of Null-BDL and H19-BDL mouse livers (*n* = 5 mice/group). **c**, **d** qPCR of the expression of pri-let-7a-1/7d/7f-1 (**c**) and pre-let-7a-1/7d/7f-1 (**d**) in the cytoplasmic and nuclear extracts of control (con) and maternal *H19* knockout (HKO) mouse BDL livers (*n* = 5 mice/group). **e** qPCR of the expression of let-7a-5p and let-7d-5p in Huh7 cells transfected with the indicated plasmids for 48 h. Data were shown as mean ± SEM from triplicate assays. N.S. no significance; *P < 0.05, **P < 0.01

We isolated the nuclear and cytoplasmic fractions of BDL livers and determined the subcellular levels of the pri-let-7a-1/7d/7f-1 transcript and let-7 precursors. The subcellular fractionation efficiency was determined by the quantification of Rnu6 and Snord68 (Fig. S2B and S2C). H19 overexpression reduced pri-let-7a-1/7d/7f-1, pre-let-7a-1 (precursor of let-7a-5p), pre-let-7d (precursor of let-7d-5p), and pre-7f-1 (precursor of let-7f-5p) in nuclear fractions of BDL livers; the cytoplasmic levels of the three precursors were not significantly affected by H19 overexpression (Figs. 3a, b). Under H19-deficiency condition, more pri-let-7a-1/7d/7f-1, pre-let-7a-1, pre-let-7d, and pre-7f-1 accumulated in nuclear fractions of BDL livers; in addition, cytoplasmic pre-let-7d but not pre-7a-1 and pre-7f-1 increased after H19 knockout (Figs. 3c, d). Without subcellular fractionation, we were unable to detect significant differences of pri-let-7a-1/7d/7f-1, pre-let-7a-1, pre-let-7d, and pre-7f-1 between H19-BDL and Null-BDL livers and unable to discriminate significant changes of pri-let-7a-1/7d/7f-1 between HKO-BDL and con-BDL livers; however, the three precursors were significantly upregulated by H19-deficiency (HKO-BDL vs Con-BDL) (Fig. S2D and S2E). When normalized to Rnu6 or Snord68, similar to the un-normalized, we observed the same alteration trends of these fractionated preforms in both H19-BDL and HKO-BDL livers, although not significant in some cases (Fig. S2F-S2I). These results suggest that H19 facilitates the processing of mature let-7 miRNAs from their preforms. Indeed, when exogenous pri-let-7a-1 or pri-let-7d were transfected into Huh7 cells, their mature forms were elevated by H19 overexpression, confirming that H19 promoted the maturation of let-7 miRNAs (Fig. 3e).

### PTBP1 physically interacts with pre-let-7a-1 and pre-let-7d

The biogenesis of let-7 is subjected to the regulations at genomic, transcriptional, and posttranscriptional levels^35^. To date, several RBPs, which recognize let-7 precursors and affect their processing have been characterized. For instance, Lin-28 homology B (Lin-28B) serves as a classic suppressor of let-7 biogenesis via binding to their precursors^17^. PTPB1 has been identified as an interacting partner of H19^8^ and is involved in RNA processing, transport, and metabolism through a direct association with target RNAs^12,36^.

**Fig. 4:**
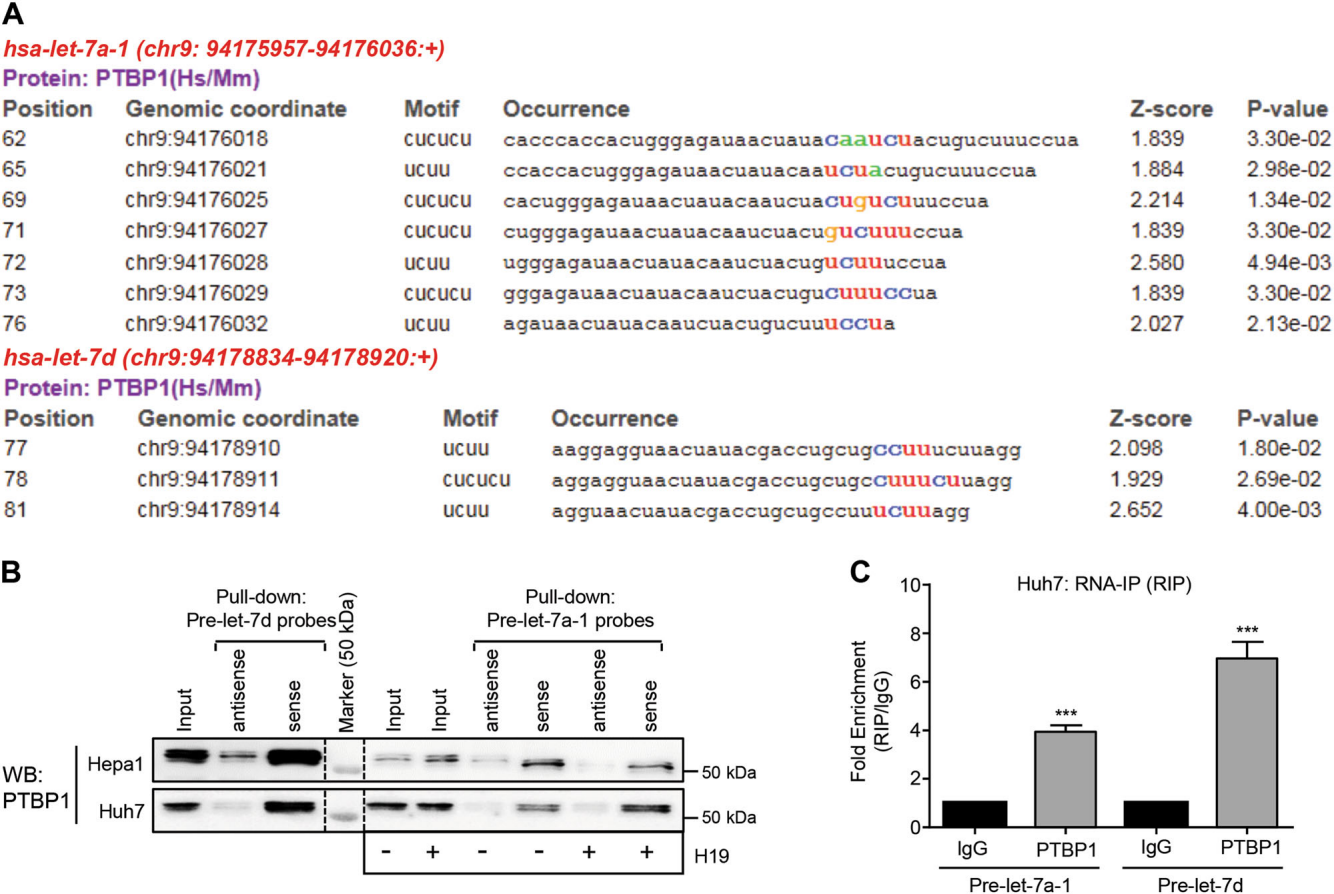
Polypyrimidine tract-binding protein 1 (PTBP1) physically interacts with pre-let-7a-1/7d. **a** Predicated PTBP1-binding sites in the human pre-let-7a-1/7d using RBPmap (http://rbpmap.technion.ac.il). The position, genomic motifs, occurrence, *z*-score, and *P*-value were shown. **b** RNA pull-down followed by western blotting. Either cell lysates of Hepa1 or Huh7 or cell lysates prepared from Hepa1 or Huh7 cells transfected without (−) or with (+) H19 overexpression plasmid were incubated with biotin-labeled pre-let-7d (sense or antisense) or pre-let-7a-1 (sense or antisense) probes. After pull down, the recruited PTBP1 to probes was examined by western blotting with anti-PTBP1 antibody. **c** RNA-immunoprecipitation (RIP) assay to detect the interactions between PTBP1 and pre-let-7a-1 or pre-let-7d using anti-PTBP1 or immunoglobulin GIgG (negative control) in Huh7 cells. Data were shown as mean ± SEM from triplicate assays. ***P < 0.001

To determine whether PTBP1 participates in the biogenesis of let-7 miRNAs, online bioinformatics tools (RBPmap and starBase v2.0) were employed to predict PTBP1-binding motifs in the stem-loop sequences of both mouse and human let-7a-1, let-7f-1, and let-7d (they are clustered in the same chromosome). RBPmap analysis revealed that there were multiple potential PTBP1-binding motifs within pre-let-7a-1 and pre-let-7d but not within pre-let-7f-1 in both humans and mice (Fig. 4a and Fig. S3).

Biotinylated RNA pull-down assays with RNA probes of pre-let-7a-1 and pre-let-7d were performed to examine the interactions between PTBP1 and pre-let-7a-1/pre-let-7d in human Huh7 and mouse Hepa1 cell lines. The results showed that PTBP1 physically interacted with pre-let-7a-1 and pre-let-7d in both cell lines (Fig. 4b). Pre-let-7a-1 probes recruited more PTPB1 in H19 overexpression (+) Huh7 cells than control (−) cells, suggesting that H19 facilitated the association of PTBP1 with pre-let-7a-1 (Fig. 4b). Of note, the different sizes of PTBP1 detected in the pull-down assays were due to post-translational phosphorylation and/or acetylation modifications^37^. Reciprocally, immunoprecipitation (RIP) assays using PTPB1 antibody further revealed the interactions between PTBP1 and pre-let-7a-1/pre-let-7d in Huh7 cells (Fig. 4c).

### H19 negatively regulates PTBP1 expression in mouse cholestatic livers

**Fig. 5:**
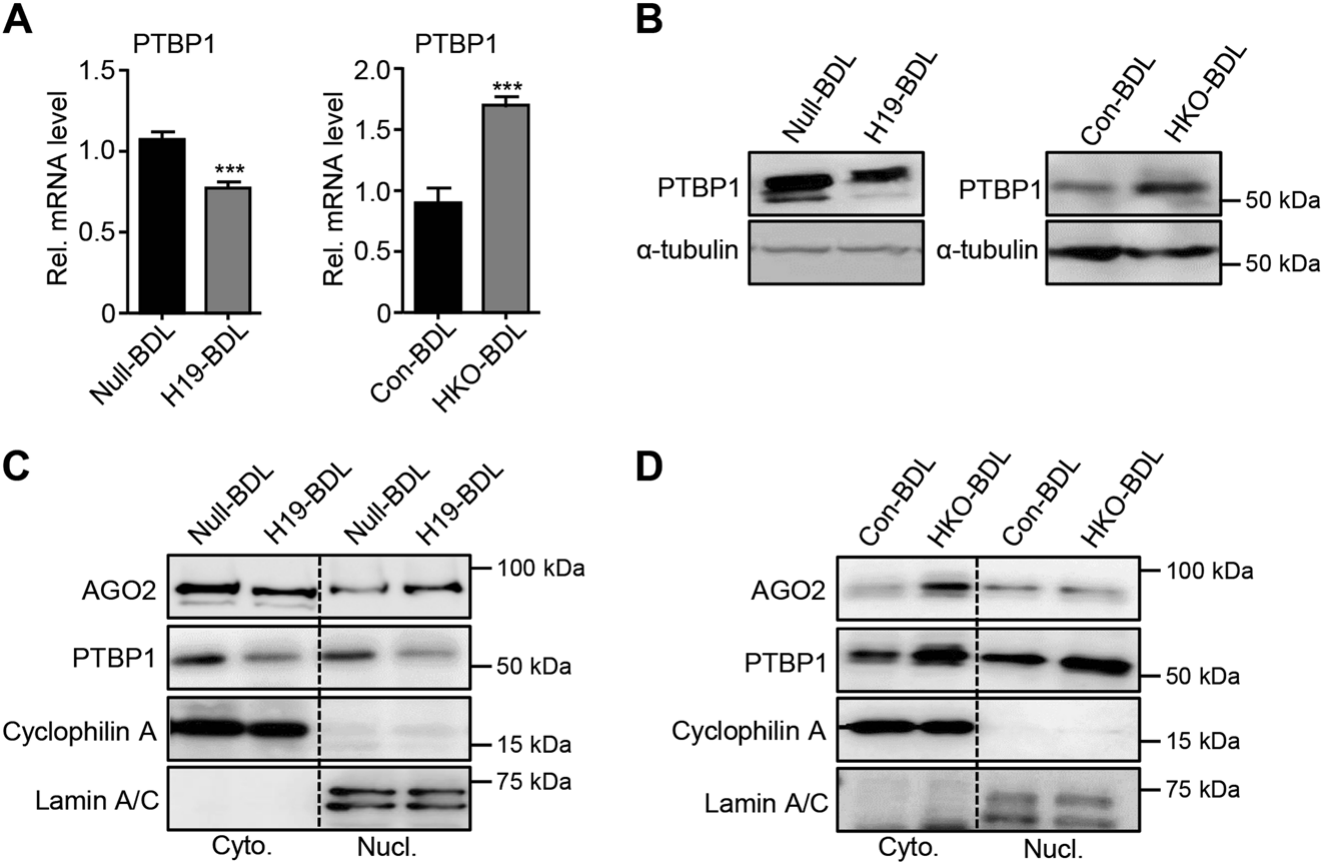
H19 decreases polypyrimidine tract-binding protein 1 (PTBP1) expression in cholestasis. (**a**) Quantitative PCR of PTBP1 expression in control and H19-overexpression or maternal *H19* knockout bile duct ligation (BDL) mouse livers. Left, Null-BDL vs H19-BDL; right, Con-BDL vs HKO-BDL. Data were shown as mean ± SEM from triplicate assays (*n* = 5 mice/group). ***P < 0.001. **b** Western blotting of PTBP1 in mouse livers as **a**. **c**, **d** Western blotting of the expression of indicated proteins in cytoplasm and nucleus fractions prepared from BDL mouse livers. Cyclophilin A was used as a cytoplasmic fraction maker and Lamin A/C was used as a nuclear fraction marker. **c**, **d** Protein samples were pooled from five individual mice per group

Next, we examined whether H19 affected PTBP1 expression in BDL-induced cholestasis. qPCR results demonstrated that PTPB1 mRNA was significantly decreased in H19-BDL livers vs Null-BDL livers, and increased in HKO-BDL livers vs Con-BDL livers (Fig. 5a). Similarly, PTBP1 protein levels were downregulated by H19 overexpression and upregulated by H19 depletion (Fig. 5b). Corresponding to the induction of H19 in cholestasis, we examined PTBP1 mRNA expression in BA-insulted cells in vitro and found that it was significantly decreased by the treatments with several BAs in MSC cells (Fig. S4). In addition, CDCA reduced PTBP1 mRNA in hepatocytes, whereas UDCA decreased PTBP1 mRNA in MLC cells.

The subcellular shuttling between the cytoplasm and nucleus is critical to PTBP1’s function^38^. In H19-BDL livers, PTBP1 protein was reduced in both cytoplasmic and nuclear fractions in comparison with Null-BDL livers (Fig. 5c). The simultaneous increase of PTBP1 protein in both subcellular fractions was found in H19-deficient cholestatic (HKO-BDL) livers (Fig. 5d). Interestingly, the indispensable miRNA processing factor protein AGO2 (also named as RISC catalytic component) decreased in cytoplasmic fractions but accumulated in nuclear fractions of H19-BDL livers (Fig. 5c); in addition, AGO2 was remarkably increased in the cytoplasmic fractions of HKO-BDL livers (Fig. 5d). These suggest that H19 might dampen the function of RISC through inhibiting AGO2 to regulate miRNA bioavailability. Overall, these findings indicated that H19 suppressed PTBP1 expression without altering its subcellular localization in cholestatic mouse livers.

### H19/PTBP1 modulates the expression and bioavailability of let-7a-5p/7d-5p in vitro

**Fig. 6:**
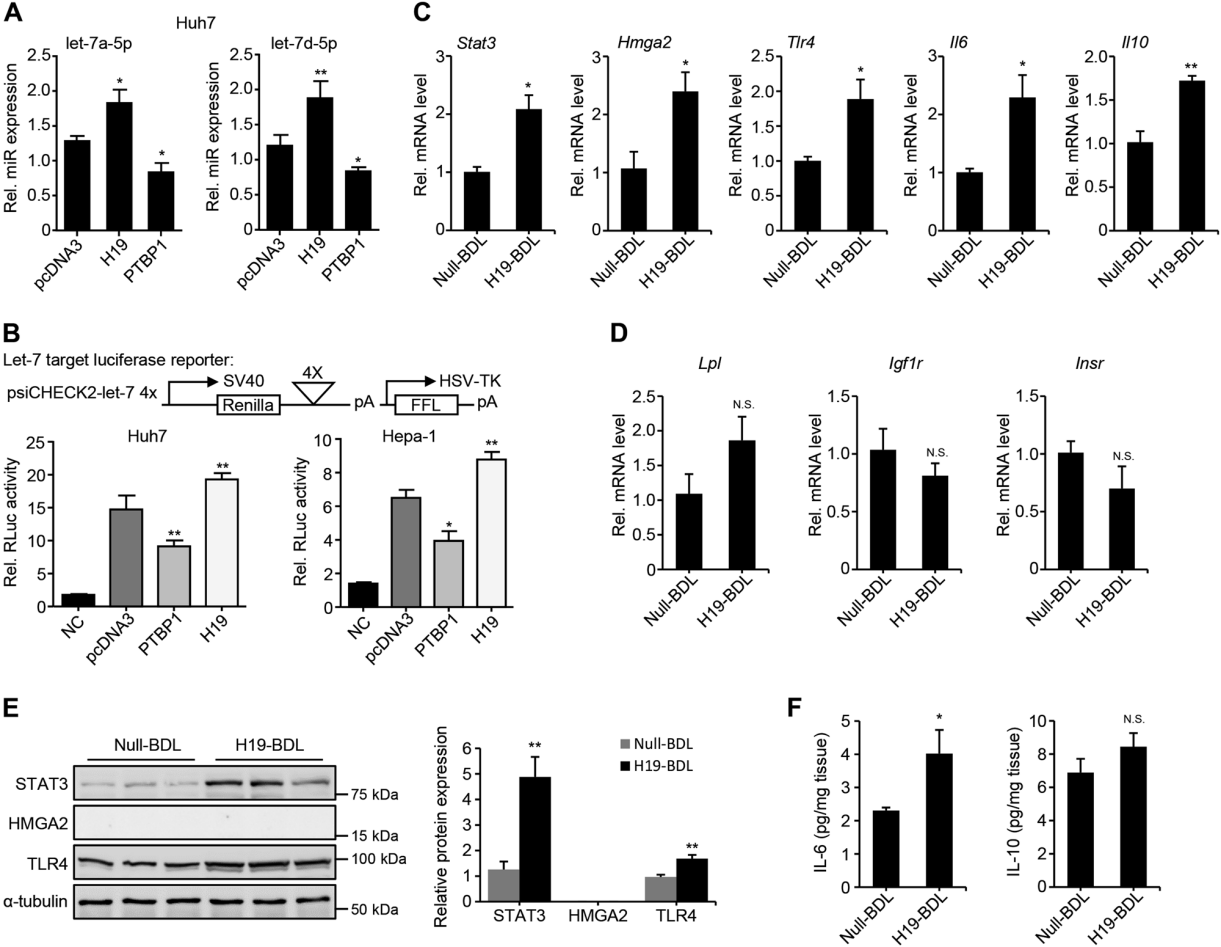
H19/PTBP1 modulates the expression and bioavailability of let-7a-5p/7d-5p in vitro. **a** Quantitative PCR (qPCR) of let-7a-5p and let-7d-5p in Huh7 cells transfected with pcDNA3, H19, or polypyrimidine tract-binding protein 1 (PTBP1) plasmids for 48 h. **b** Luciferase reporter assays using psiCHECK2-let-7 4× (top) in Huh7 (left) and Hepa1 (right) cells transfected with pcDNA3, PTBP1, and H19 plasmids. NC none transfected. The renillar luciferase (RLuc) activity was determined 24 h after transfection. **c**, **d** qPCR of let-7 target genes in Null-BDL and H19-BDL mouse livers as Fig. 1c. **e** Western blotting of indicated proteins in Null-BDL and H19-BDL mouse livers (left). Protein samples were from three individual mice per group. The band densitometry was quantified using ImageJ and relative protein expression was graphed (right). **f** Enzyme-linked immunosorbent assay of interleukin (IL)-6 and IL-10 in Null-BDL and H19-BDL mouse livers (*n* = 5 mice/group). N.S. no significance; *P < 0.05, **P < 0.01

To further understand the regulation of let-7 miRNAs by H19/PTBP1, we selected let-7a-5p/7d-5p as two representative miRNAs to further examine the effects of H19/PTBP1 on the expression of let-7 miRNAs in vitro. H19 overexpression in Huh7 cells dramatically increased the levels of let-7a-5p and let-7d-5p, while PTBP1 overexpression had a suppressive effect on their levels (Fig. 6a).

Functionally, we used let-7 biosensor psiCHECK2-let-7 4×, which contains four let-7-binding sites downstream of the renilla luciferase (Rluc) coding region, to determine how H19/PTBP1 regulates let-7 miRNAs’ bioavailability. Two HCC cell lines Huh7 and Hepa1, both of which contained detectable levels of let-7a-5p/7d-5p, were transfected with psiCHECK2-let-7 4× together with empty pcDNA3, PTBP1, or H19 plasmid. The inhibition of let-7a-5p/7d-5p on Rluc activity was confirmed via co-transfection of psiCHEK2-let-7 4× and let-7a-5p/7d-5p (Fig. S5). The Rluc activity was decreased after PTBP1 overexpression, indicating that PTBP1 facilitated let-7a-5p and let-7d-5p to target their binding sites within Rluc mRNA and inhibited Rluc expression. In contrast, H19 overexpression prevented let-7a-5p/7d-5p from binding to Rluc mRNA and increased Rluc activity as compared with the negative control (pcDNA3) (Fig. 6b). These results are consistent with the previous finding that H19 serves as a “Sponge” of let-7 to restrain its bioavailability^26^.

Therefore, these findings suggested that H19 increased let-7a-5p/7d-5p production, with an inhibitory effect on the bioavailability to their targets, and that PTBP1 decreased let-7a-5p/7d-5p production and promoted their bioavailability. We examined the expression levels of several let-7 targets to interrogate the functional significance of H19 regulation of let-7 in BDL-induced cholestatic livers, considering the opposite effects of H19 on let-7 expression and bioavailability. The hepatic mRNA expression of several inflammation-related target genes of let-7, including *Stat3*^39^, *Hmga2*^40,41^, *Tlr4*^42^, *Il6*^43^, and *Il10*^43^ significantly increased in H19-BDL vs Null-BDL livers (Fig. 6c), suggesting H19 might decrease let-7 bioavailability to exacerbate BDL-induced hepatic inflammatory response, which is in concordance with the previous finding that H19-BDL livers develop more severe liver injury and fibrosis than Null-BDL livers^7^. In contrast, the hepatic mRNA expression of metabolism-related let-7 target genes *Lpl*^44^, *Igf1r*^45^, and *Insr*^45^ did not show significant change after H19 overexpression (Fig. 6d). H19-BDL livers displayed more protein expression of STAT3 and TLR4, with an undetectable level of HMGA2 revealed by western blotting (Fig. 6e). Further, ELISA revealed that IL-6 but not IL-10 increased in H19-BDL vs Null-BDL livers (Fig. 6f).

## Discussion

**Fig. 7:**
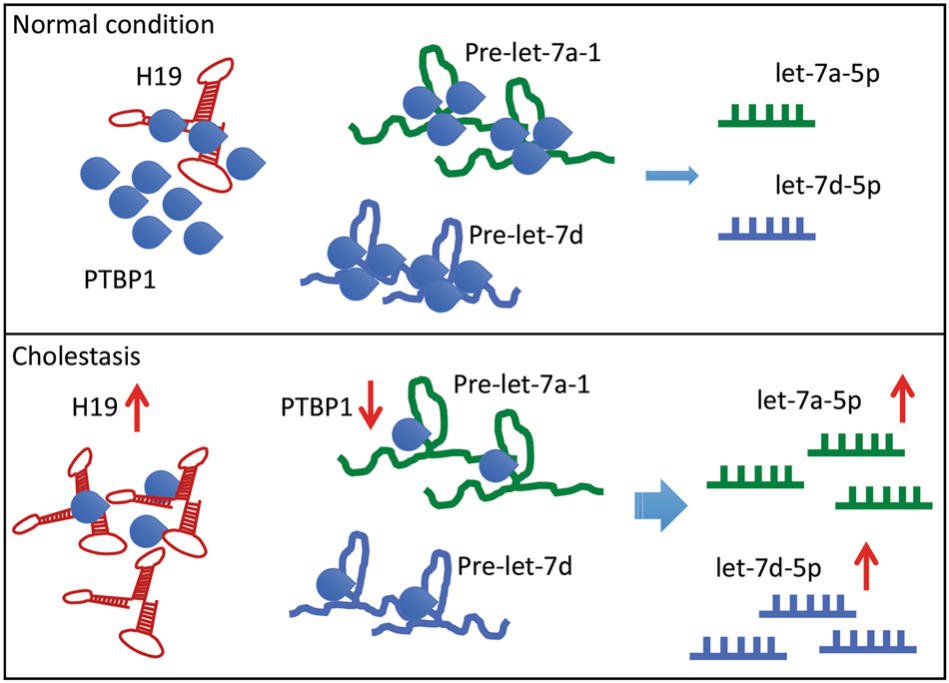
Schematic of H19/PTBP1 regulation of let-7a-5p/7d-5p expression in cholestasis. Under a normal condition, polypyrimidine tract-binding protein 1 (PTBP1) interacts with H19 and is also incorporated into the precursors of let-7a-1 and let-7d. The binding of PTPB1 to precursors of let-7 might prevent the biogenesis of mature let-7 miRNAs. During cholestasis, H19 expression is activated, which leads to the reduction of PTPB1. Concomitantly, the suppressive effect of PTPB1 on the processing of pre-let-7a-1 and pre-let-7d is alleviated. Therefore, the production of let-7a-5p and let-7d-5p is potentiated

The dysregulation of let-7 miRNAs is found in cholestatic liver disorders^19,46^. In this study, we reveal that lncRNA H19 promotes the biogenesis and expression of a cluster of let-7 miRNAs, including let-7a-5p, let-7d-5p, and let-7f-5p, in cholestatic mouse livers, and functionally suppresses their bioavailability. We also find that H19 antagonizes the expression of PTBP1, an H19-interacting protein, which associates with pre-let-7d and pre-let-7a-1 and inhibits let-7 biogenesis but promotes their bioavailability (Fig. 7).

Because of the hydrophobic or hydrophilic nature as well as the different affinity to BA receptors, BAs act as signaling molecules but display differential potency to regulate gene expression^47^. We find that the hydrophilic UDCA significantly and to the maximal extent increases the expression of let-7a-5p/7d-5p/7f-5p in primary hepatocytes but almost has no effect in MLC and MSC cells (Fig. 2). A previous study shows that H19 is only induced by LCA among several BAs in primary hepatocytes^7^. The inconsistent induction of H19 and let-7 miRNAs by UDCA in primary hepatocytes suggest that UDCA may upregulate let-7a-5p/7d-5p/7f-5p in hepatocytes independent of H19. Indeed, H19 is mainly expressed in cholangiocytes and cholangiocyte-derived exosomes, which are the sources of H19 in hepatocytes during cholestatic disease progression^48^. We show that LCA moderately decreases let-7a-5p and let-7d-5p in primary hepatocytes, which apparently disputes a positive correlation between H19 and let-7 miRNAs expression. Thus, isolating primary hepatocytes from cholestatic livers may help to establish a positive expression correlation between let-7 and H19 in hepatocytes. Indeed, in primary hepatocytes isolated from cholestatic mouse livers, the expression of let-7a-5p/7d-5p/7f-5p was enhanced by H19 overexpression (Fig. S1C). Furthermore, CA, TCA, or both induces the expression of let-7a-5p/7d-5p/7f-5p in MSC and MLC cells (Fig. 2b, c), which supports a positive correlation between H19 and let-7 miRNA expression.

We show that the hydrophobic BA CDCA, the most efficacious endogenous FXR ligand, which is in low level or absent in cholangiocytes^49^, decreases the expression of let-7a-5p/7d-5p/7f-5p in MSC and MLC cells, suggesting that let-7 expression can be induced independent of FXR. In vitro cultured MSC and MLC cells might not well reflect the precise regulation of let-7 expression by H19 in vivo. Therefore, isolating primary cholangiocytes from cholestatic livers to examine let-7 and H19 levels will be the future directions. Further, the dysregulated levels of let-7a-5p/7d-5p/7f-5p in H19-modulated cholestatic mouse livers is a consequence of multiple additive/synergistic effects of H19 not only on BA components but also on other biological processes, such as intrahepatic inflammation and biliary proliferation.

It has been shown that PTBP1 is in complex with AGO2 and miRNAs and is involved in let-7-loaded miRNA-induced silencing complex in human cells, indicating potential roles of PTBP1 in miRNA-mediated gene regulation^50^. In this study, for the first time, we identify PTBP1 as a novel pre-let-7d/pre-let-7a-1 interacting partner, which expands the regulatory network of let-7 biogenesis. The binding of PTBP1 to pre-let-7 might reduce their accessibility to Dicer and/or degrade pre-let-7 directly by recruiting certain RNases or miRNAs, which have been used as mechanisms by other RBPs, such as LIN28A/B and hnRNP A1, to post-transcriptionally regulate let-7 biogenesis^17,25,51^. The exact mechanisms by which PTPB1 suppresses let-7 biogenesis need further investigation.

Under pathological conditions, the association between RBPs and RNAs can be dynamically enhanced or disturbed. We find that H19 significantly induces let-7a-5p/7d-5p/7f-5p in cholestatic mouse livers and facilitates the interactions between PTBP1 and pre-let-7d/pre-let-7–7a-1 in vitro, suggesting that the PTBP1/let-7 miRNAs may be involved in the progression of cholestasis. A handful of studies show that let-7 family members are engaged in several biliary diseases. For example, let-7i is able to modulate lipopolysaccharide receptor Toll-like receptor (TLR) expression in inflammation processes in cholangiocytes^52^. Inhibition of let-7a in BDL mice increases intrahepatic bile duct mass and the expression of nerve growth factor^53^. Let-7d is downregulated in primary biliary cirrhosis (PBC) liver tissues compared with normal tissues in human^54^. Let-7b is significantly decreased in the peripheral blood cells of PBC patients^55^. Therefore, we will continue to determine the association between PTBP1 and pre-let-7d/pre-let-7a-1 in the BDL mouse model and reveal their expression correlation in human cholestatic liver diseases, which will shed light on the molecular mechanisms of pathogenesis and progression of human cholestatic liver diseases.

There are still some open questions: (1) are H19, PTBP1, and pre-let-7 integrated into the same complex? (2) If so, what are other functional factors including proteins and RNAs? (3) Although H19 associates with PTBP1 and reduces its mRNA and protein expression, it is unknown whether H19 regulates PTBP1 transcriptionally or post-transcriptionally. (4) The interaction between H19 and pre-let-7 needs to be elucidated. (5) The intriguing part of this study is that H19 potentiates let-7 expression but decreases its bioavailability. The “Sponge” function of H19 to restrain let-7 bioavailability has been revealed^15,26,44^. We find that H19 decreases the cytoplasmic level of AGO2, an essential component of the RISC to silence gene expression; this may also partially explain the upregulation of let-7 target genes in H19-BDL livers (Fig. 6). The biological significance of let-7 miRNA upregulation in cholestatic livers needs further investigation. It is unknown whether their upregulation by H19 is a feedback to dampen H19’ pathological effects through inhibiting let-7 target genes, which are upregulated by H19.

A pri-let-7a-1/7d/7f-1 transcript from the locus on mouse chromosome 13 can produce a high percentage of let-7a-5p, let-7d-5p, and let-7f-5p. However, let-7a-5p can also originate from chromosome 9 and let-7f-5p can be derived from chromosome X. We show that PTBP1 interacts with pre-let-7a-1 and pre-let-7d, and decreases let-7a-5p and let-7d-5p (Figs. 4 and 6). However, pre-let-7f-1 potentially does not have any PTBP1 binding motif. It still needs to determine whether PTBP1 affects the let-7f-5p level.

H19 functions via physically associating with its partners. Nuclear H19 controls a series of Imprinted Gene Network genes to modulate embryo growth through functional interaction with methyl-CpG-binding domain protein 1 to establish H3K9me3 repressive markers at their DMRs^1^. Cytoplasmic H19 can bind to a RBP, human antigen R, and prohibit miR-675 processing to limit the growth of placenta before birth^56^. The implication of H19/PTBP1 in regulating let-7 expression and bioavailability may help to delineate the molecular mechanisms of let-7 dysregulation in various human diseases.

## Supplementary information

Supplementary Figure 1

Supplementary Figure 2

Supplementary Figure 2 continued

Supplementary Figure 3

Supplementary Figure 3 continued

Supplementary Figure 4

Supplementary Figure 5

Supplementary Table 1

Supplementary Table 2

Supplementary EXCEL file
