# Supplementary figures and images for: H19 potentiates let-7 family expression through reducing PTBP1 binding to their precursors in cholestasis
Source: Cell Death Dis. 2019 Feb 18;10(3):168. doi: 10.1038/s41419-019-1423-6 (PMC6379488; doi:10.1038/s41419-019-1423-6)

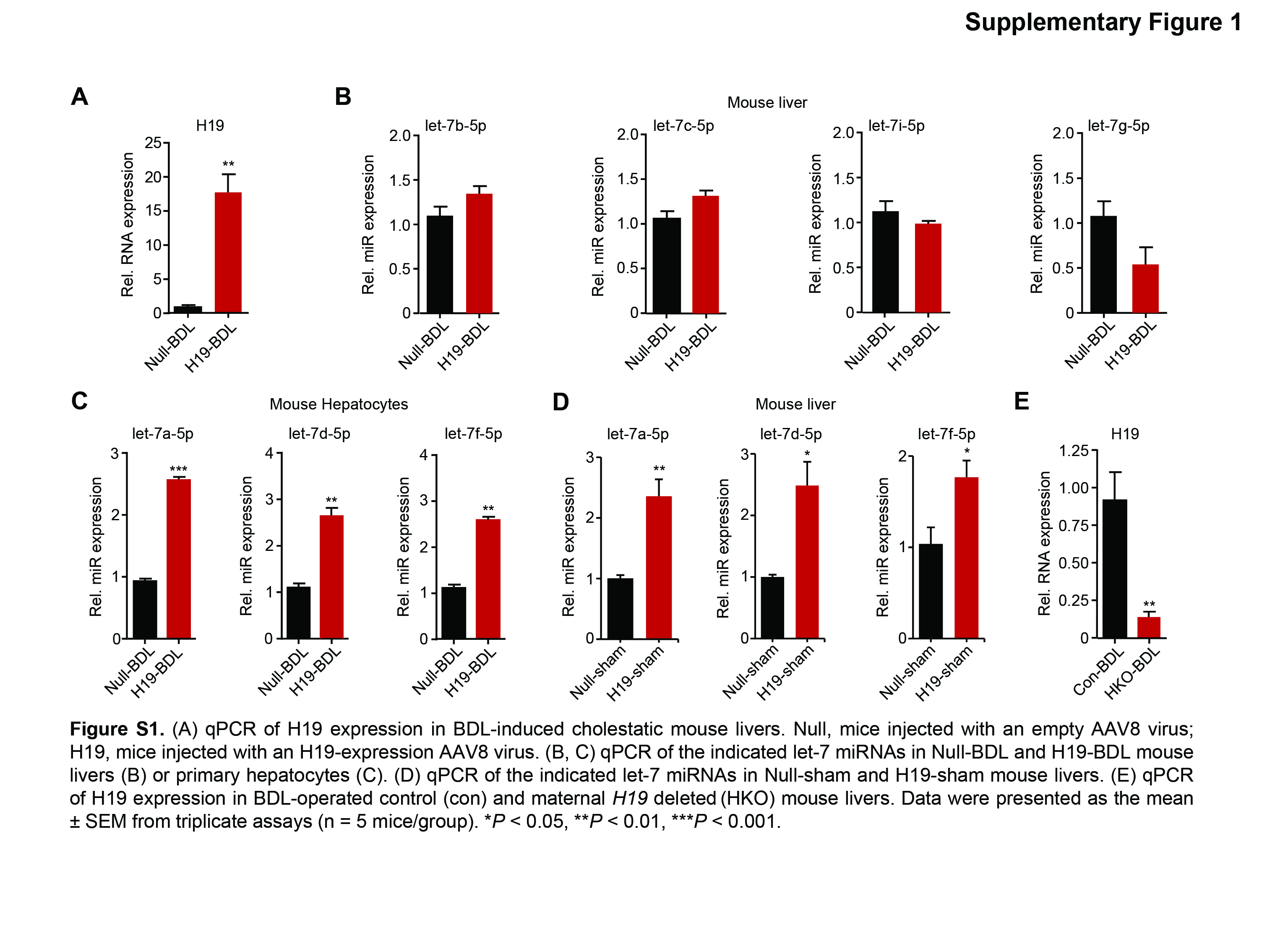

Supplement: Supplementary file 1 — Supplementary Figure 1 [file 41419_2019_1423_MOESM1_ESM.jpg]

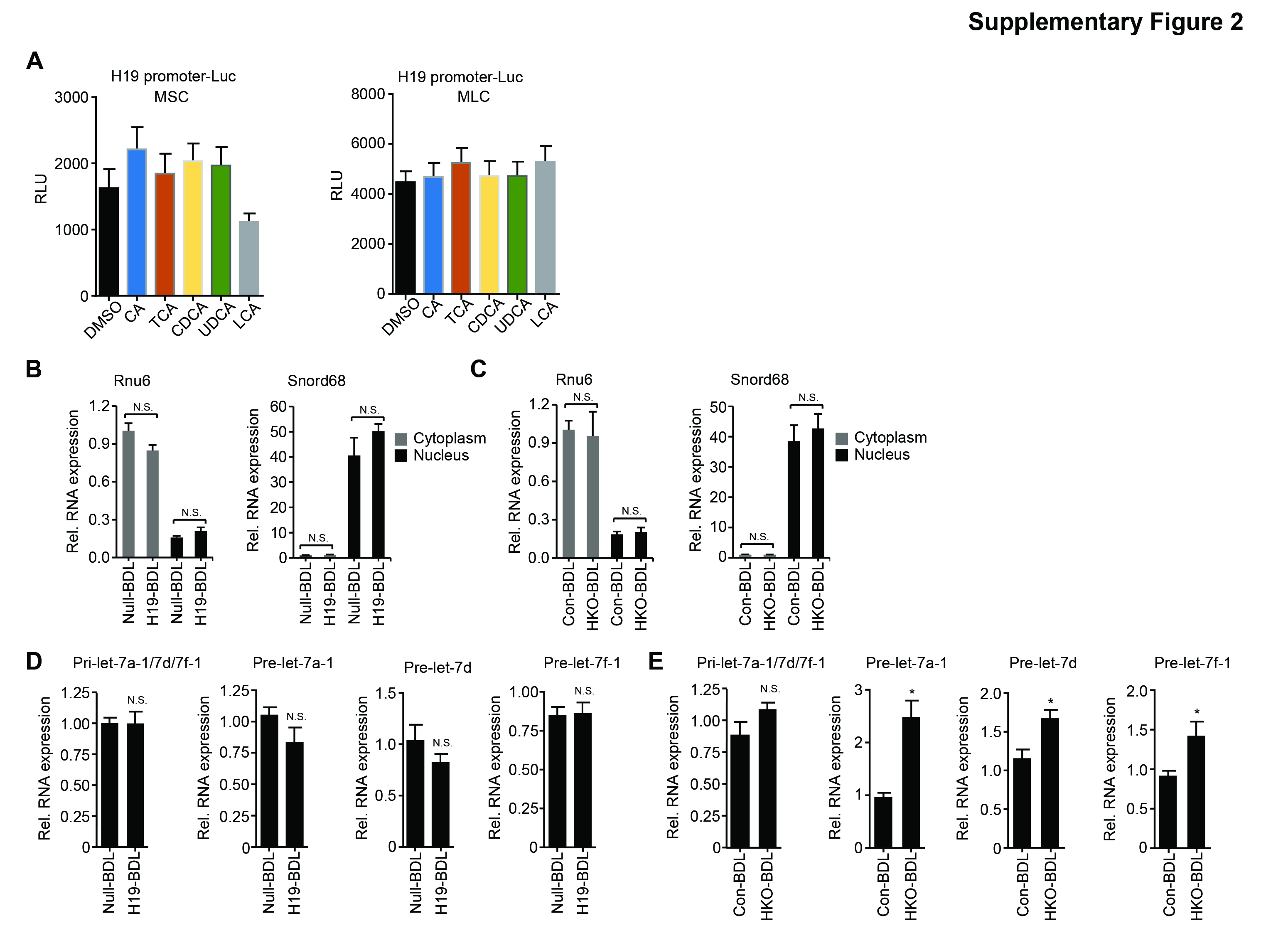

Supplement: Supplementary file 2 — Supplementary Figure 2 [file 41419_2019_1423_MOESM2_ESM.jpg]

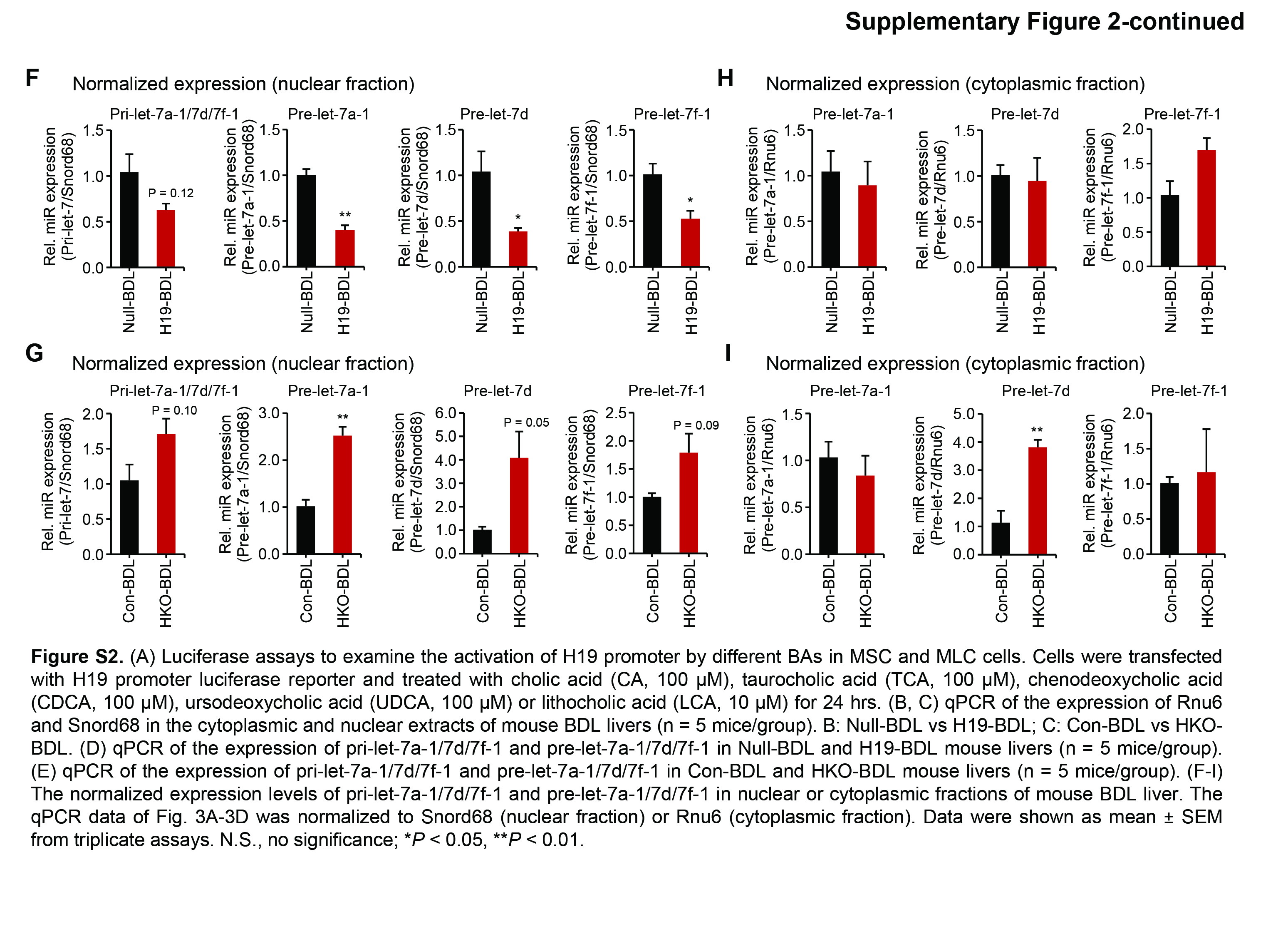

Supplement: Supplementary file 3 — Supplementary Figure 2 continued [file 41419_2019_1423_MOESM3_ESM.jpg]

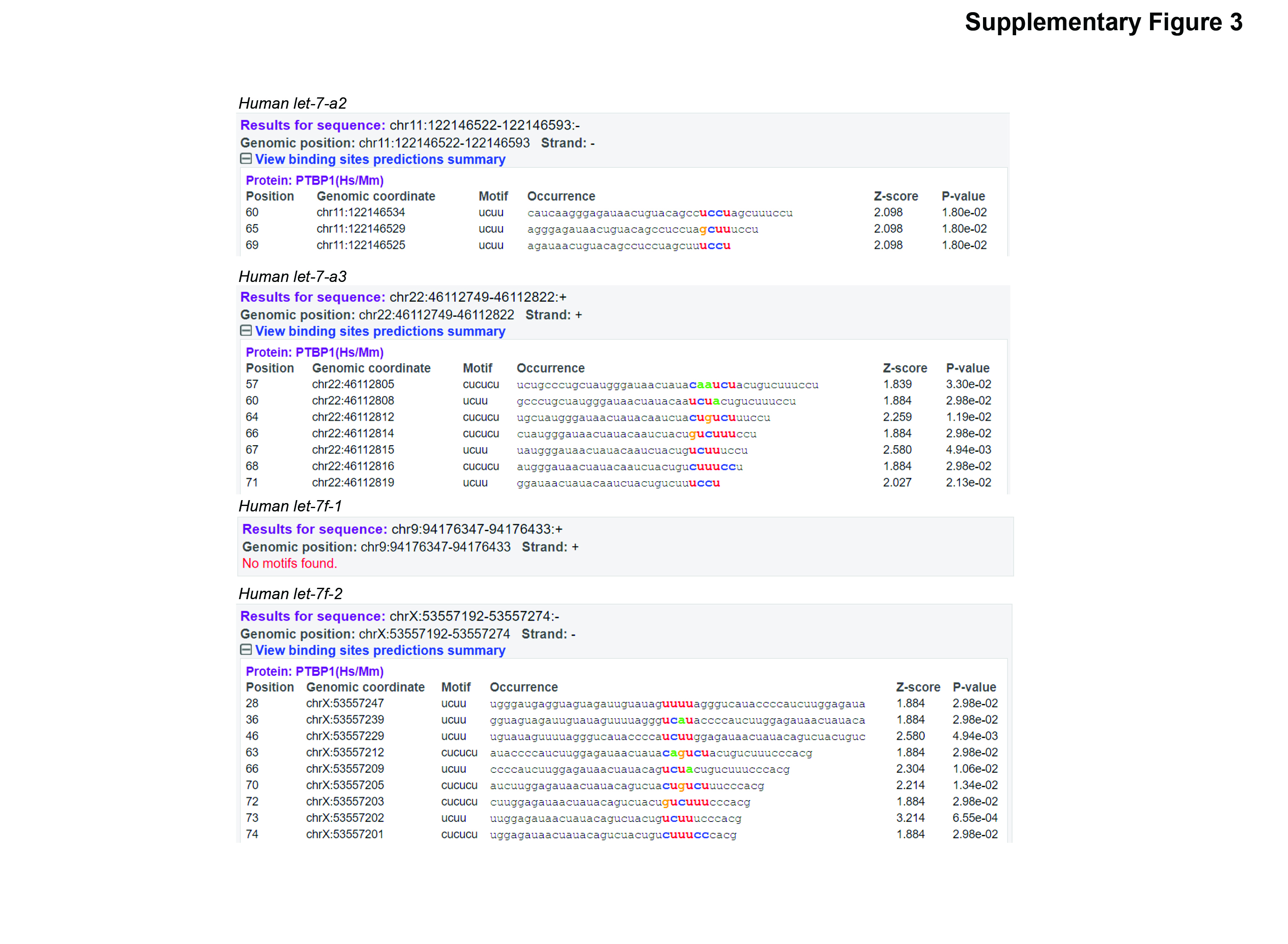

Supplement: Supplementary file 4 — Supplementary Figure 3 [file 41419_2019_1423_MOESM4_ESM.jpg]

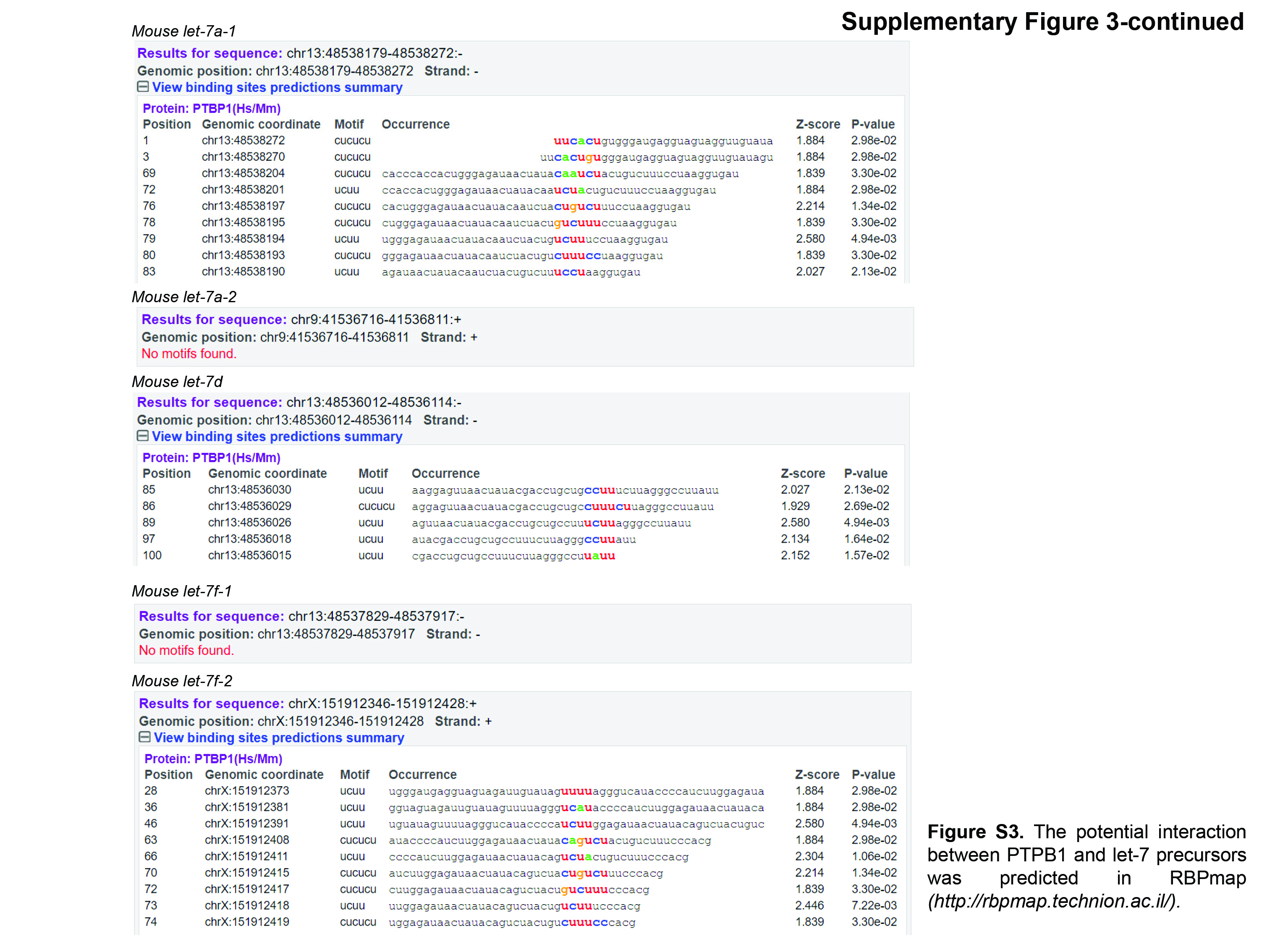

Supplement: Supplementary file 5 — Supplementary Figure 3 continued [file 41419_2019_1423_MOESM5_ESM.jpg]

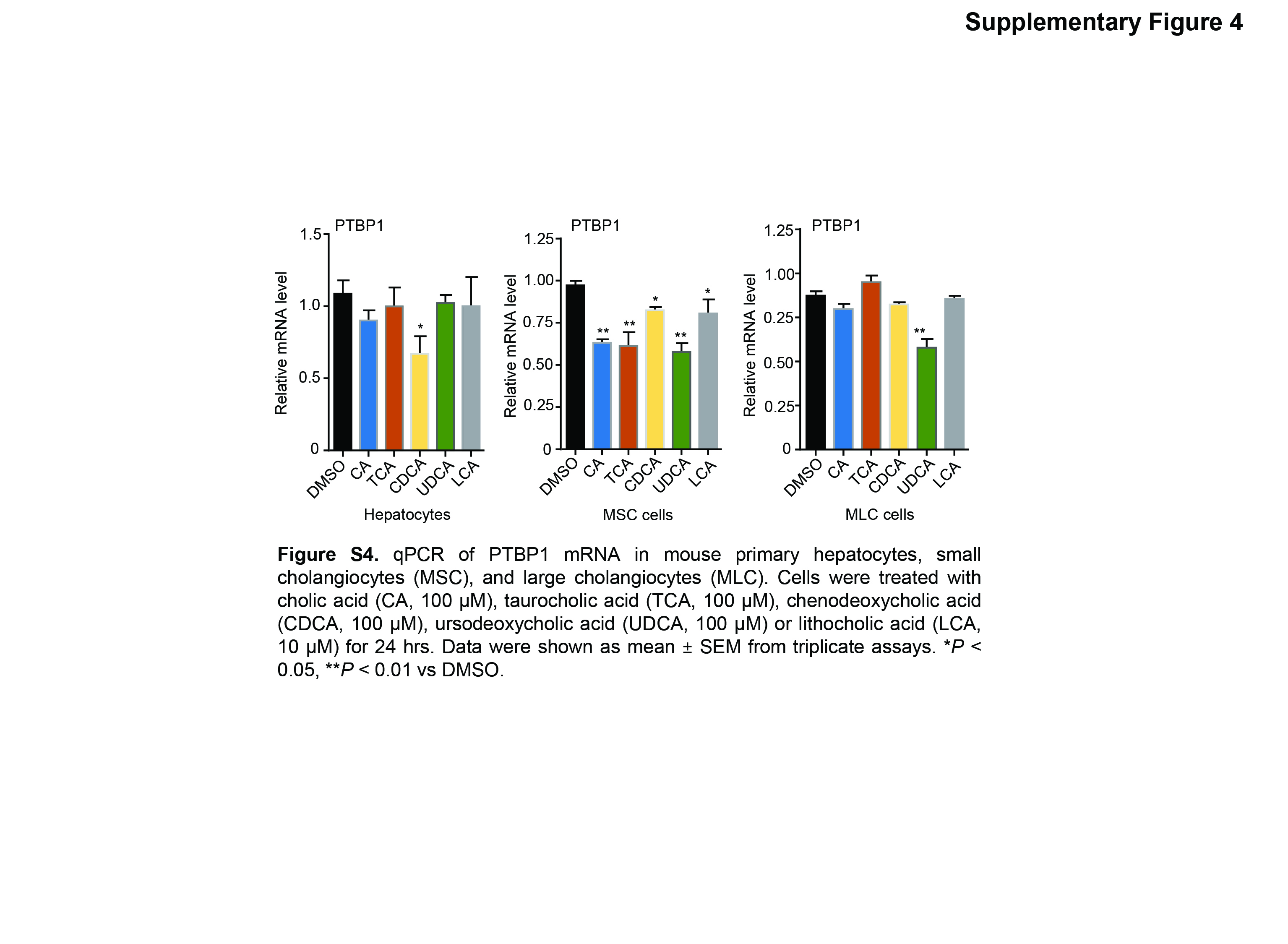

Supplement: Supplementary file 6 — Supplementary Figure 4 [file 41419_2019_1423_MOESM6_ESM.jpg]

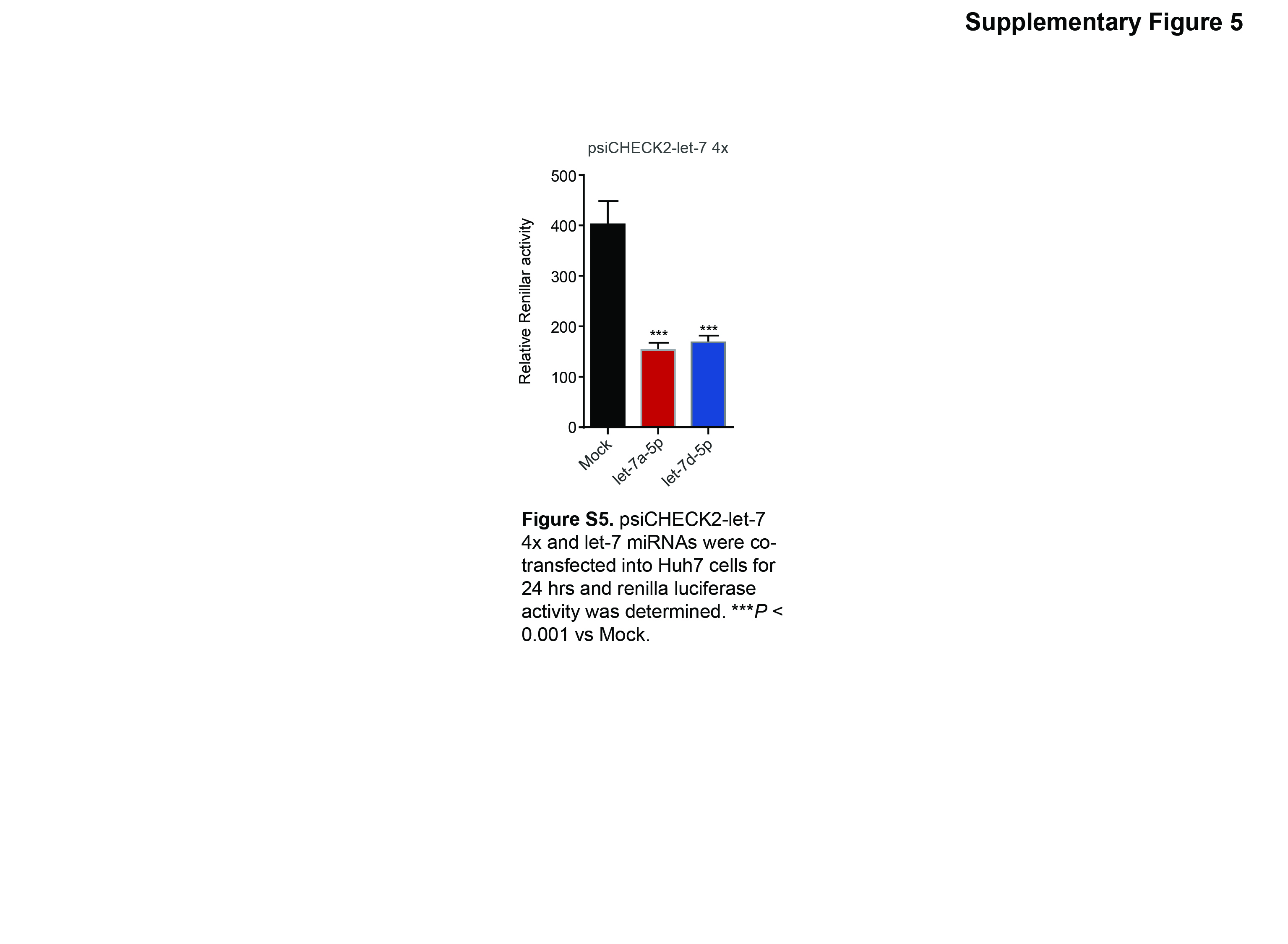

Supplement: Supplementary file 7 — Supplementary Figure 5 [file 41419_2019_1423_MOESM7_ESM.jpg]

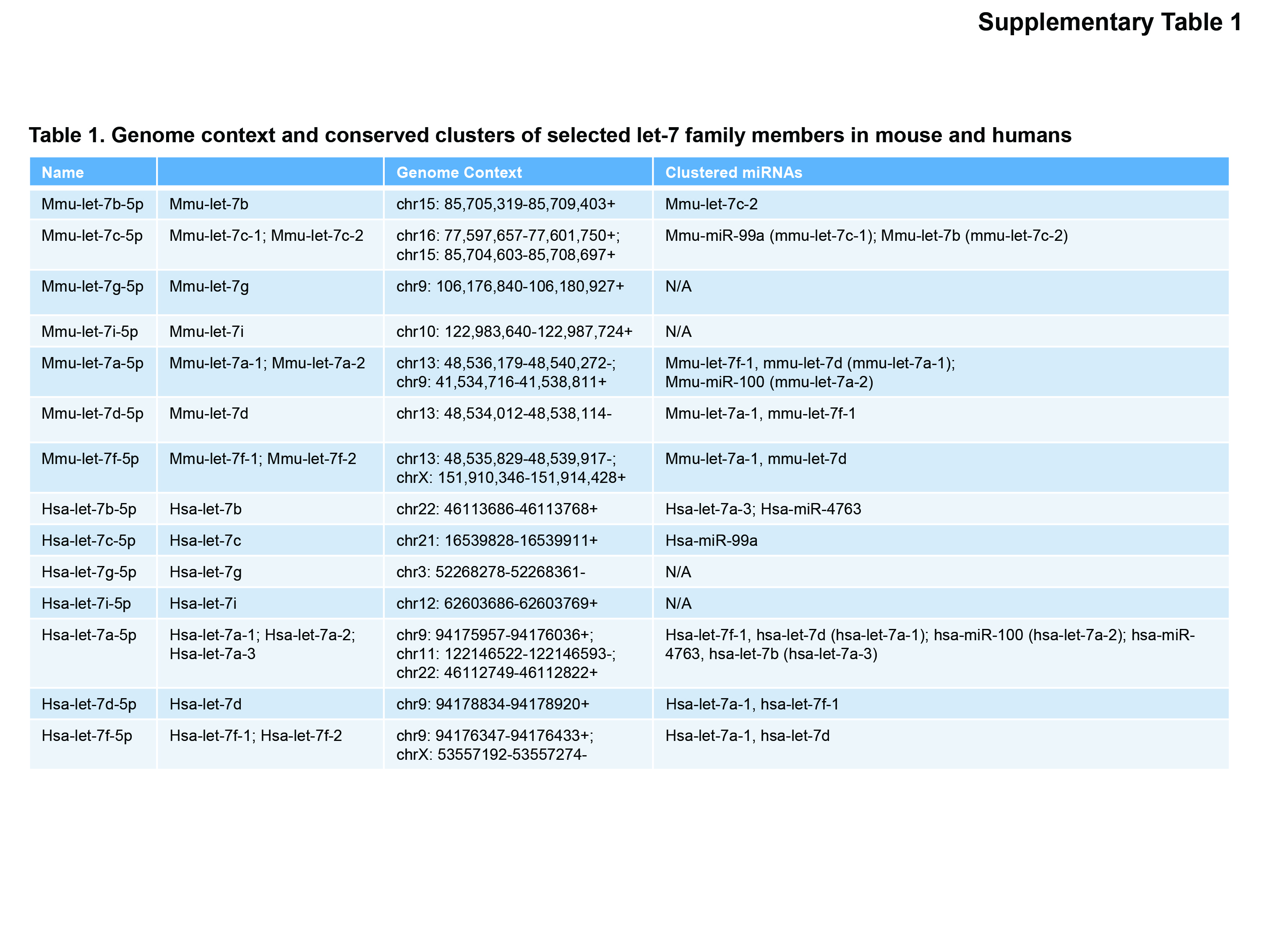

Supplement: Supplementary file 8 — Supplementary Table 1 [file 41419_2019_1423_MOESM8_ESM.jpg]

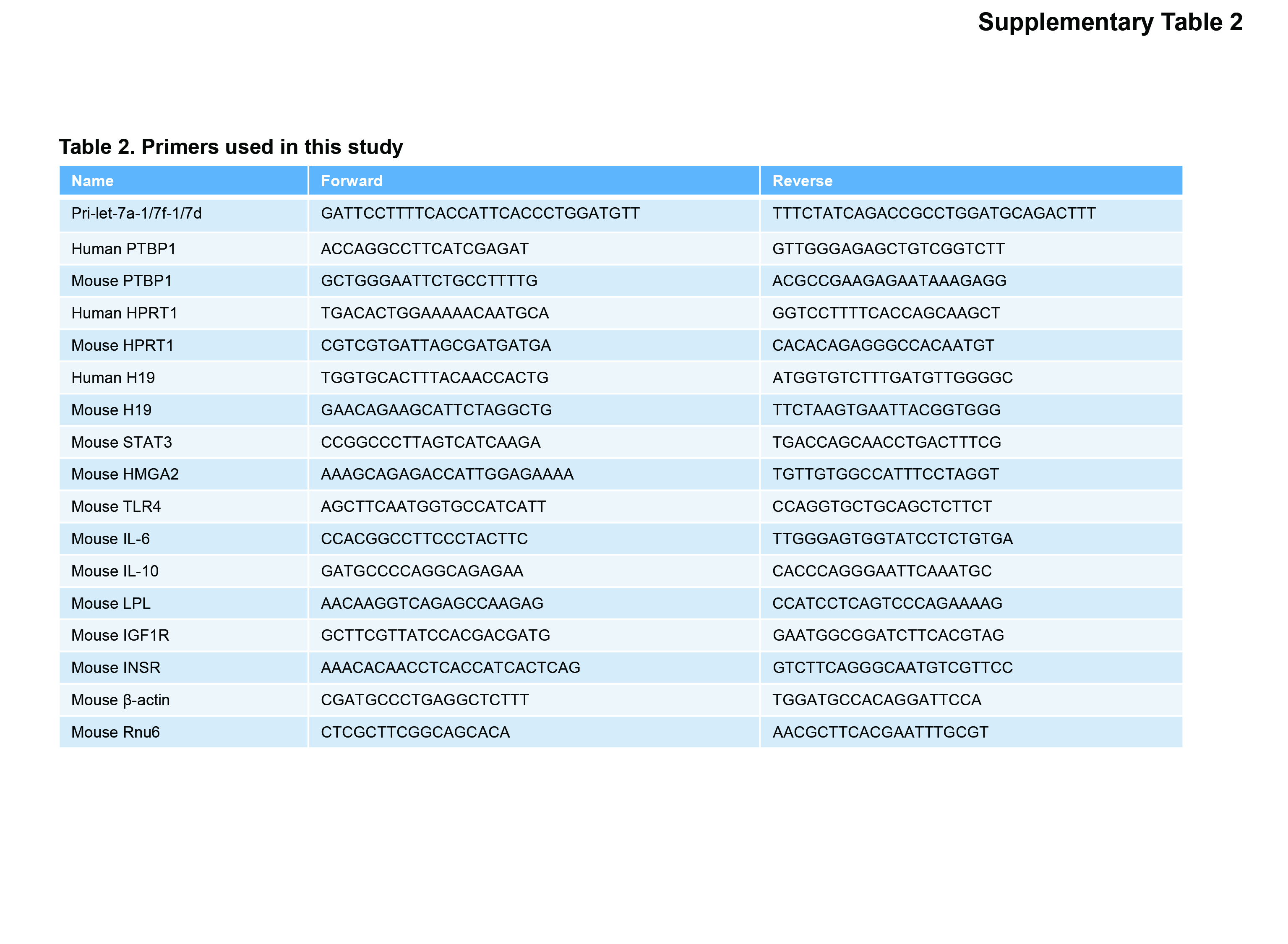

Supplement: Supplementary file 9 — Supplementary Table 2 [file 41419_2019_1423_MOESM9_ESM.jpg]
